# Dummy Templated Receptors Showing Enhanced Affinity for Vitamin D3

**DOI:** 10.3390/molecules31010001

**Published:** 2025-12-19

**Authors:** Abed Abdel Qader, Musa I. El-Barghouthi, Börje Sellergren, Ali I. Ismail, Lubna Alrawashdeh, Talah Salman, Moh’d Moahand Ahmad Al-Dabet, Eman Zmaily Dahmash

**Affiliations:** 1 Department of Chemistry, Faculty of Science, Isra University, P.O. Box 22, Amman 11622, Jordan; 2 Department of Chemistry, Faculty of Science, The Hashemite University, P.O. Box 330127, Zarqa 13133, Jordan; musab@hu.edu.jo (M.I.E.-B.); ali.ismail@hu.edu.jo (A.I.I.); lubna.reyad@hu.edu.jo (L.A.); 3 Department of Biomedical Sciences, Faculty of Health and Society, Malmö University, 20506 Malmö, Sweden; borje.sellergren@mau.se; 4 Faculty of Pharmacy, Isra University, Amman 11622, Jordan; talahsalman@gmail.com (T.S.); eman.zmaily@iu.edu.jo (E.Z.D.); 5 Department of Medical Laboratories, Faculty of Health Sciences, American University of Madaba (AUM), Amman 11821, Jordan; m.dabet@aum.edu.jo; 6 Department of Chemical and Pharmaceutical Sciences, School of Life Sciences, Pharmacy and Chemistry, Kingston University, London KT1 2EE, UK

**Keywords:** molecularly imprinted polymers, vitamin D3, template–monomer interactions, vinyl pyridine, computational chemistry

## Abstract

Vitamin D_3_ (VD3) is an essential micronutrient, but its analytical determination in biological matrices is often hindered by structurally related metabolites and the limited selectivity of conventional analytical sorbents. The preparation of a molecularly imprinted polymer (MIP) using VD3 as a template is challenging due to its hydrophobic structure and lack of polar groups. Therefore, in this work, MIPs were prepared using the closely related structure hyodeoxycholic acid methyl ester as a template and tested for their adsorption capacity toward VD3. Several MIPs were first prepared using different functional monomers, and the results showed that 4-vinylpyridine (4VP) monomer in combination with divinylbenzene (DVB) as a crosslinker exhibited a relatively high binding capacity and imprinting factor. UV spectroscopy indicated an optimal VD3–monomer ratio of 1:4, while computational modeling further confirmed favorable interactions between VD3 and 4VP. The effect of incorporating styrene as a co-monomer with 4VP was also investigated, showing an enhancement in adsorption capacity with a slight increase in the imprinting factor. However, TGA analysis revealed that the thermal stability of the MIPs decreased with higher styrene content. Overall, the prepared MIPs demonstrated improved selectivity and recognition of VD3 compared to the non-imprinted polymers, offering a promising approach for its selective extraction and quantification.

## 1. Introduction

Vitamin D (VD) is used both as a dietary supplement and in the treatment of deficiency-related conditions [1,2,3,4]. It is available in various pharmaceutical dosage forms, including tablets, capsules, oral drops, intravenous injections, and topical dosage forms as creams or ointments [5]. It is considered an essential element for all ages, specifically in the pediatric population, due to its vital role in bone mineralization and the maintenance of bone integrity [6,7]. VD deficiency (VDD) has been linked to dental issues, obesity, cardiovascular disease, and insulin resistance [1,7,8].

The importance of VD was further highlighted by the observed correlation between VD levels and the severity of COVID-19 infection [9]. The two main forms of VD are VD2 (ergocalciferol), sourced from plants, and VD3 (cholecalciferol), found in animal-based sources and produced in the skin when exposed to sunlight. VD3 is generally preferred in pharmaceutical formulations due to its higher bioavailability and stability, allowing it to be administered in lower doses [10].

Accurate measurement of VD levels is crucial due to their wide-ranging health implications. Although serum VD analysis is routine, current methods lack standardization, leading to inter-laboratory variability. Furthermore, structurally similar VD metabolites can interfere with these analyses [9,11]. VD analysis typically involves pre-treating blood samples, often using protein precipitation, liquid–liquid extraction, solid-phase extraction, or a combination thereof [12,13,14]. Furthermore, the quantification of VD is based on two key approaches: the immunoassay and chromatographic methods [15,16,17]. Despite the popularity of immunoanalytical methods, they lack the specificity of the employed antibodies, and significant interference of various metabolites can be observed [15]. On the other hand, chromatographic methods offer better separation and quantification of similar metabolites but are complex and time-consuming [9,18,19,20].

Solid-phase extraction (SPE) is a widely used technique for concentrating and purifying analytes in biological and environmental samples due to its efficiency and speed [21]. However, common sorbents often lack selectivity and may be affected by interference from similar compounds. Sorbents based on molecularly imprinted polymers (MIPs) offer a potential solution by providing high selectivity [22,23,24,25]. MIPs are synthetic polymers created by crosslinking in the presence of a template molecule. After removing the template, the polymer selectively binds to the template or similar compounds. This selective recognition is achieved through shape complementarity and various non-covalent interactions, enabling the discrimination of analytes based on shape and chemical properties [22,26,27,28].

The preparation of an MIP for VD3 is challenging due to its large hydrophobic structure, reactive double bonds, and lack of polar groups that can effectively interact with the monomers used in MIPs. To overcome this, Kia et al. prepared an MIP for VD3 using silica-based sol–gel processes [29] and electropolymerization [30] instead of the traditional bulk polymerization method. A recent interesting study explored the use of a methyl methacrylate derivative of VD3 as a functional monomer to create an MIP for detecting VD3, taking advantage of the strong affinity of the monomer for VD3 during polymerization. The resulting polymer was used as a sensor that measures VD3 concentration through electrochemical impedance spectroscopy. The sensor demonstrated an excellent limit of detection and high selectivity for vitamin D3 [31].

**Figure 1:**
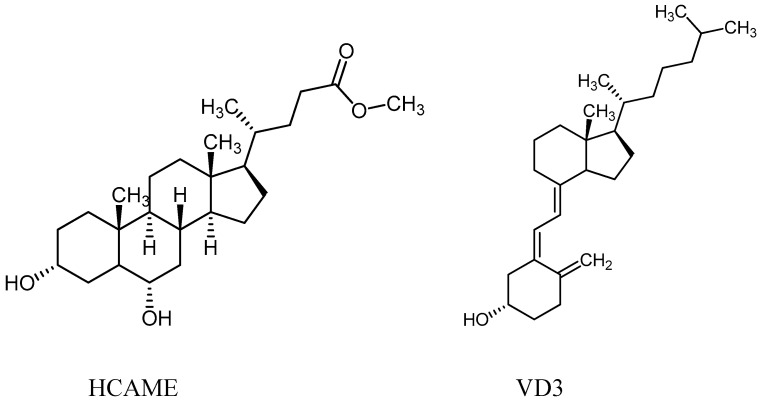
Structures of VD3 and HCAME.

In this study, we prepared MIPs for VD3 using bulk polymerization, utilizing hyodeoxycholic acid methyl ester (HCAME, Figure 1) as a template. The size of HCAME is fairly close to VD3 and possesses functional groups that can interact with the functional monomers used in MIPs. Screening of different functional monomers and two crosslinkers, as well as evaluation of the efficiency of the produced MIPs, will be carried out to assess their selectivity and binding affinity toward VD3. Our results show that the MIP prepared using 4-vinylpyridine (4VP) and divinylbenzene (DVB) as crosslinkers is the most selective for VD3; therefore, the interactions of 4VP monomers with HCAME and VD3 will be studied computationally using the PM3 method and molecular docking. Further, the interaction of VD3 with different amounts of 4VP will be studied using UV spectroscopy. The effect of adding styrene on the selectivity of the prepared MIP will also be addressed. TGA and SEM will be carried out to characterize the prepared 4VP MIP.

## 2. Results and Discussion

**Figure 2:**
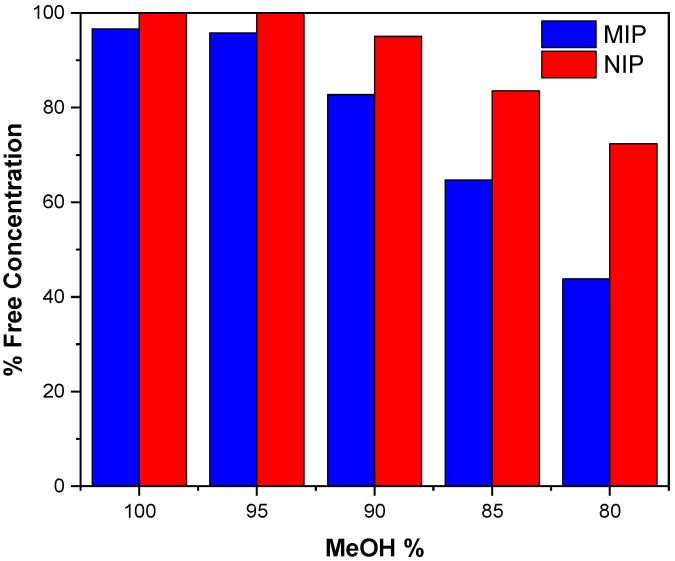
Effect of methanol percentage on the rebinding of VD3 by MIP and NIP prepared using 4VP and EGDMA.

First, MeOH was used as the medium for carrying out the binding experiments of VD3 with the MIP prepared using 4VP as a monomer, HCAME as a template and EGDMA as a crosslinker, as detailed in the experimental section, because MeOH is commonly used to precipitate proteins during the pre-treatment of blood samples [32]. However, results (Figure 2), show that at 100% MeOH, high percentages of VD3 remained in the solution, indicating its limited binding with the polymers. VD3 is poorly soluble in pure water; therefore, to reduce the affinity of VD3 to the medium and enhance its binding to the polymers, various compositions of MeOH/water ranging from 100% to 80% (by volume) were tested. At 95% to 90% MeOH, a slight reduction in free VD3 concentration was observed, indicating a minor increase in its affinity to MIP. When the MeOH percentage was further reduced to 85% and 80%, the binding efficiency of the MIP improved significantly, resulting in a pronounced decrease in free VD3 concentration; therefore, 80% MeOH was used a as a binding medium for all experiments.

### 2.1. Screening of Functional Monomers

**Figure 3:**
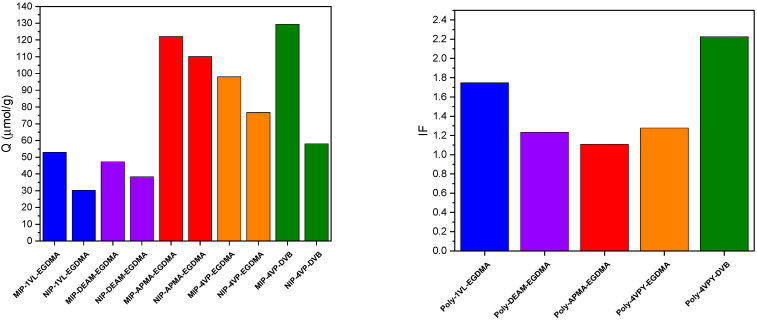
Binding capacity (Q, (**left**)) and imprinting factor (IF, (**right**)) for VD3 by MIPs.

A series of MIPs using HCAME as a template and their corresponding NIPs were prepared using different monomers, including 1VL, DEAM, APMA, and 4VP, with EGDMA as the crosslinker. Further, MIP using 4VP as a monomer and DVB as a crosslinker was also prepared. The adsorption capacities (Q) for VD3 were measured and are presented in Figure 3. The results show among the synthesized MIPs, those prepared using 4VP and APMA monomers exhibit the highest adsorption capacities, while those prepared with 1VL and DEAMA show the lowest. This may be attributed to the fact that 4VP and APMA are hydrogen bond donors and possess aromatic rings, enabling them to interact more effectively with the target analyte through hydrogen bonding and dispersion interactions. However, the results clearly demonstrate that the selectivity of the prepared MIPs is low, as indicated by the values of the estimated imprinting factor (IF), which are in the range of ~1.1–1.3, except for the 1VL polymer, which shows a relatively higher IF (~1.7); nevertheless, its low adsorption capacity (Q) limits its use. On the other hand, MIP prepared using DVB as a crosslinker exhibits an increased Q value and a significant enhancement in selectivity (IF ≈ 2.3). We should stress here that the MIPs used in the binding experiments conducted in this section were only dried and crushed, without being sieved prior to the binding experiments.

### 2.2. UV-Vis Titrations

**Figure 4:**
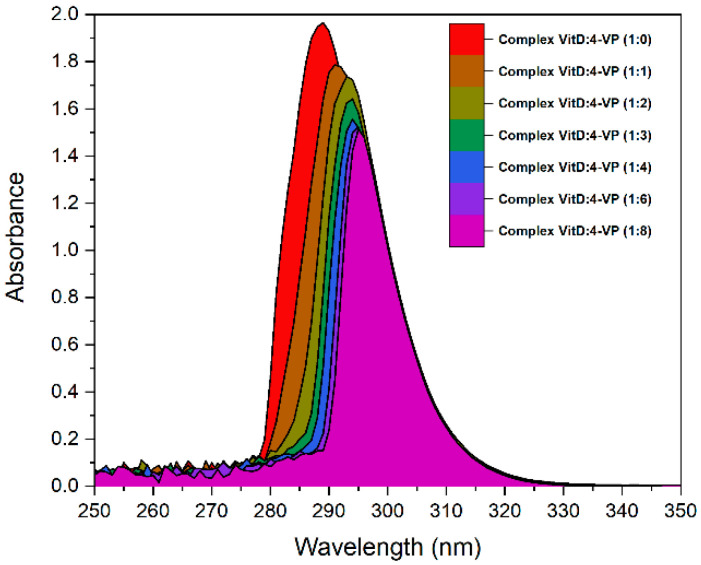
The difference UV-Vis absorption spectra of 1.0 mM VD3 at different concentrations of 4VP carried out in methanol–water (80:20, *v*/*v*) mixture and 25 °C.

Based on the above results, it is clear that the best candidate for the monomer is 4VP, and that for the crosslinker is DVB. To rationalize the optimal VD3–monomer ratio, the interactions between VD3 and the 4VP monomer were studied by recording the difference UV-Vis absorption spectra in the absence and presence of various VD_3_–monomer ratios (Figure 4). A gradual decrease in peak intensity, accompanied by a red shift of the peak maximum, is observed with increasing monomer concentration, most likely due to non-covalent interactions between VD3 and the monomer. These changes appear to be most pronounced up to a 1:4 VD3–monomer ratio; beyond this ratio, changes are still observable, but to a much lesser extent. These results suggest that four molecules of 4VP may be sufficient to surround and interact strongly with one molecule of VD3. Therefore, this ratio will be used in preparation of the MIP based on HCAME as a template and 4VP as a monomer.

### 2.3. MIPs Using 4VP and 2VP Monomers

**Figure 5:**
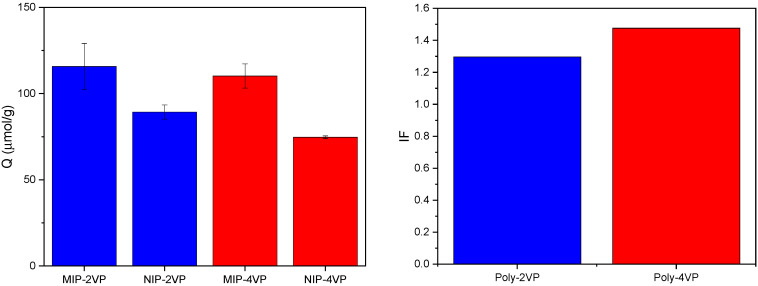
Adsorption capacity (Q) and imprinting factors (IF) values for 4VP-DVP and 2VP-DVP polymers.

Figure 5 shows the adsorption capacity (Q) and the imprinting factor (IF) for binding of VD3 by MIP-4VP-DVB and its corresponding NIP (both polymers were sieved to obtain particle size fractions between 32 and 45 μm), which reveal Q values of 110 and 75 μmol/g for MIP and NIP, respectively. The IF value is estimated to be ~1.45, demonstrating the selectivity of the prepared MIP toward VD3. Further, we extend this work to prepare MIP using the closely related monomer, 2VP (Figure 5), which shows a slightly higher adsorption capacity but with lower selectivity.

### 2.4. Effect of Adding Styrene

**Figure 6:**
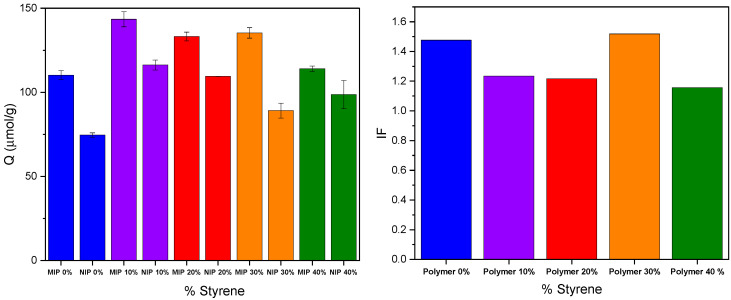
Adsorption capacity (Q, (**left**)) and imprinting factor (IF, (**right**)) of 4VP-DVB MIPs prepared with varying amounts of styrene.

Styrene is commonly used as a functional monomer in the synthesis of MIPs due to its ability to enhance binding affinity and selectivity for various target molecules through hydrophobic and π-π interactions [33,34,35,36]. For example, when combined with divinylbenzene, styrene provided effective hydrophobic binding for 1-hydroxypyrene [35]. Additionally, styrene contributed to the formation of highly selective microporous membranes for phenol when incorporated into styrene–acrylonitrile copolymer matrices [36]. Therefore, we investigated the effect of adding styrene during the preparation of the 4VP-DVP MIP on its performance (Figure 6). The results clearly show that the addition of styrene enhances the binding of VD3, most likely due to the hydrophobic nature of styrene and its influence on polymer porosity. It should be noted that this enhancement is minimal when 40% styrene is added compared to the lower percentages. The corresponding NIPs prepared in the presence of styrene also exhibit better affinity to VD3, and as a result, the IF values are slightly reduced, except for MIP with 30% added styrene, which shows a slight increase in its IF (~1.5).

### 2.5. Effect of Template-to-Monomer Ratio

**Figure 7:**
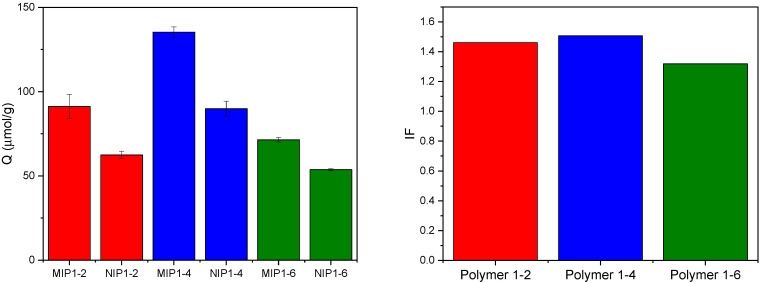
Q and IF values of MIP polymers prepared using different ratios of HCAME and 4VP.

Furthermore, the effect of template-to-monomer ratio on performance of the MIPs was studied, with all MIPs prepared with 30% styrene (Figure 7). The results clearly demonstrate that the 1:4 ratio is the optimal one, as noticed from the significant difference in the Q values of the 1:4 ratio compared to 1:2 and 1:6 ratios.

### 2.6. Adsorption Isotherms

**Figure 8:**
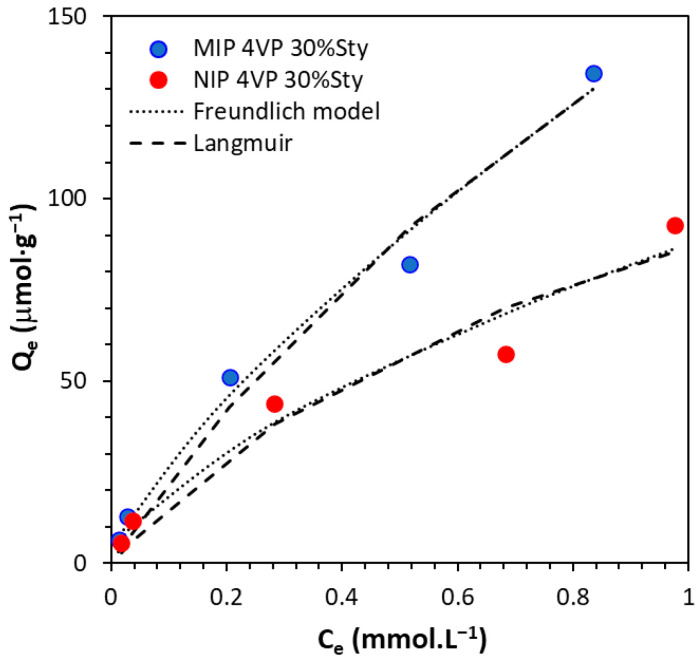
Binding isotherms for the uptake of VD3 by MIP-4VP-30%STY and its corresponding NIP in MeOH/H_2_O (80:20).

The adsorption isotherms for MIP-4VP-30%STY and its corresponding NIP, along with the Freundlich and Langmuir fittings, are shown in Figure 8. and fitting parameters are listed in Table 1. It is clear that the adsorption capacity of the MIP is higher than that of the NIP over the studied concentration range, demonstrating the effectiveness of the imprinted polymer. Langmuir fitting (which assumes monolayer adsorption) yields a Q_max_ of ~385 and 173 µmol·g^−1^ for the MIP and NIP, respectively. The residual sum of squares (RSS) and the Fisher parameters (F) were used to evaluate how well the isotherm models applied to the adsorption behaviors; typically, the isotherm model with the most fitting parameters yielded lower RSS values and higher F values. The experimental data were reasonably fitted to both models; however, the Freundlich model appears to be a better fit, as evidenced by its slightly higher R^2^, lower RSS, and higher F values, which may reflect the heterogeneous nature of the polymer surface.

### 2.7. Thermogravimetric Analysis (TGA) of MIPs with Varying Styrene Content

**Figure 9:**
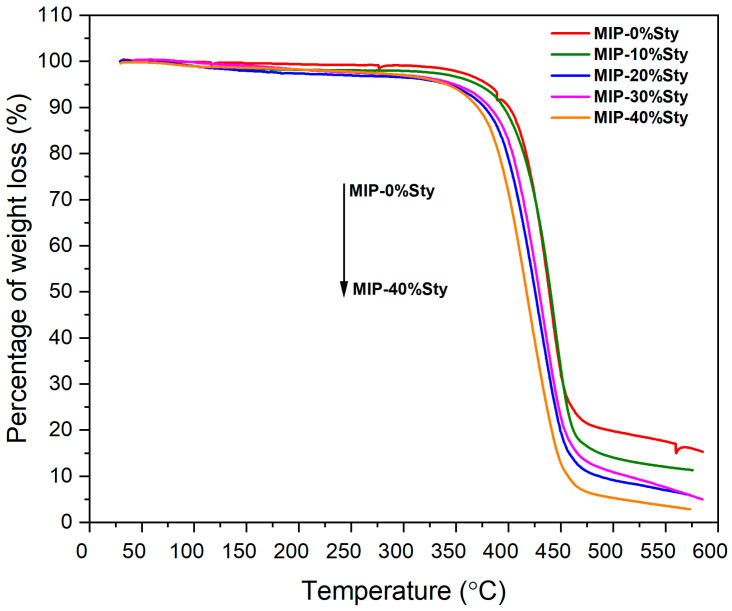
TGA curves of 4VP-MIPs with varying styrene contents.

The thermal stability of 4VP-MIPs with varying styrene contents was examined by TGA in air at 10 °C/min up to 600 °C (Figure 9). All samples show minimal weight loss below 200 °C, indicating good initial stability due to low moisture content and low-volatility components. Significant thermal degradation starts at ~300 °C, since all samples start to experience loss of weight, which probably corresponds to the breakdown of the polymer backbone and degradation of other organic components. It is clear from the curves that increasing the styrene content is accompanied by lower thermal stability, most likely because styrene-rich polymers produce more volatile degradation products. These results highlight the need to optimize polymer composition for high-temperature applications.

### 2.8. Scanning Electron Microscope (SEM)

**Figure 10:**
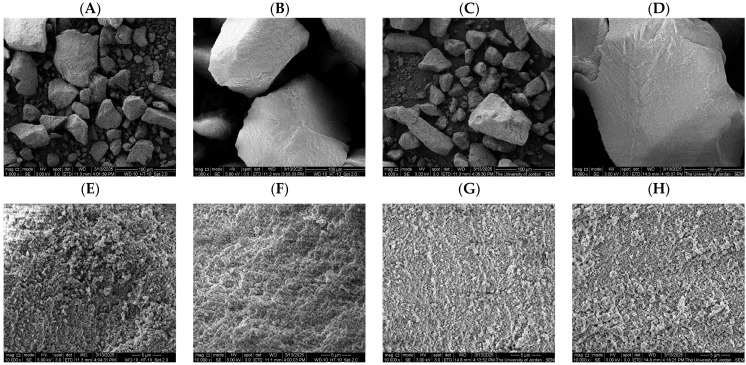
SEM images of MIP and NIP polymers synthesized with and without styrene. (**A**,**B**) MIP and NIP prepared without styrene at 100 μm magnification; (**C**,**D**) MIP and NIP prepared with styrene at 100 μm magnification; (**E**,**F**) MIP and NIP prepared without styrene at 5 μm magnification; (**G**,**H**) MIP and NIP prepared with styrene at 5 μm magnification.

The surface morphologies of MIP and NIP polymers, prepared with and without styrene, were investigated by SEM, as shown in Figure 10. Significant differences in particle size, surface texture, and porosity were observed depending on the presence of styrene as a co-monomer. In the absence of styrene (Figure 10A,B,E,F), both MIP and NIP exhibited irregular, dense, and aggregated morphologies with relatively smooth surfaces. The compact nature of these particles suggests a low degree of porosity and limited surface area, which may reduce the accessibility of binding sites. This morphology is consistent with polymerization in the absence of an effective co-monomer, resulting in poor particle dispersion and limited network organization. In contrast, the incorporation of styrene (Figure 10C,D,G,H) led to the formation of smaller, more uniform particles with rougher and more porous surfaces. At higher magnification (5 μm), the MIP (Figure 10G) and the NIP (Figure 10H) prepared with styrene look quite similar in their overall surface texture. However, the MIP (Figure 10G) shows some noticeable separate microcavities, while the NIP (Figure 10H) appears to have a generally rougher, more porous surface with no distinct microcavities. These morphological differences support the conclusion that the removal of the template during the imprinting process generates specific binding sites within the polymer matrix. The rough and porous surface structure of the MIP is particularly advantageous, as it facilitates greater accessibility of the recognition sites and enhances the potential for selective binding. Overall, these findings demonstrate that styrene plays a critical role in improving polymer morphology, particle uniformity, and porosity. The distinct structural differences between MIP and NIP confirm the success of the imprinting process and support the use of styrene-based MIPs for selective molecular recognition and adsorption applications.

### 2.9. Computational Results

**Figure 11:**
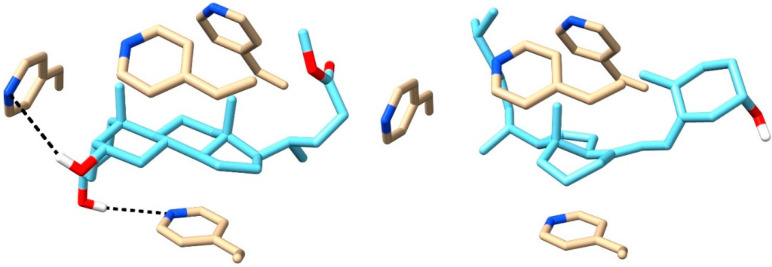
Structures of the 4VP complexes with HCAME (**left**) and VD3 (**right**).

Computational methods were used to obtain the most stable orientations of four molecules of 4VP, around the template molecule (HCAME) using the PM3 semiempirical method. The most stable configuration (Figure 11) demonstrates that two 4VP molecules form hydrogen bonds with HCAME, while the hydrophobic moieties of HCAME are mainly encapsulated within the cavity formed by the 4VP molecules. A molecular docking study of VD3, with the same spatial orientations of the four monomers obtained for the HCAME complex, revealed that the top-scoring structure displays the inclusion of VD3 within the pocket formed by the 4VP molecules, with the hydroxyl group of VD3 not involved in hydrogen bonding. Favorable interaction energies for both HCAME and VD3 with the surrounding 4VP molecules were estimated using AutoDock Vina 1.2 and the PM3 semi-empirical method (Table 2), with HCAME experiencing more favorable interactions. The results clearly indicate that the MIP polymer prepared using HCAME as the template in this study is most likely to be effective in selective binding of VD3 molecules.

## 3. Experimental

### 3.1. Materials

VD3, HCAME, styrene (STY), 4-vinyl pyridine (4VP), 2-vinyl pyridine (2VP), vinylimidazole (1Vl), 2-(Diethylamino)ethyl methacrylate (DEAM), N-(3-Aminopropyl)methacrylamide hydrochloride (APMA), divinylbenzene (DVB), Ethylene glycol dimethylacrylate (EGDMA), 2,2-azoisobutyronitrile (AIBN), acetonitrile (ACN), and dimethylsulfoxide (DMSO) were purchased from Sigma Aldrich (Darmstadt, Germany) and were used without further purification. Methanol and acetic acid were purchased for AZ Chem (Pretoria, South Africa). All other reagents were of analytical grade.

### 3.2. Methods

#### 3.2.1. HPLC

HPLC experiments were carried using a DIONEX UltiMate 3000 system (Thermo Scientific, Waldbronn, Germany) equipped with a Luna C18 column (150 mm × 4.6 mm, 5 μm, ID), protected by an RP18 guard column (4.0 mm × 3.0 mm I.D., 5 μm), both from Phenomenex (Torrance, CA, USA). The HPLC mobile phase was methanol, with a flow rate of 1 mL·min^−1^, column temperature maintained at room temperature, an injection volume of 20 μL, and a diode array detector set to 285 nm, with all compounds eluting within 10 min. Quantification was performed using external calibration peak area measurements, with linear calibration graphs obtained for VD3 in the 11–440 mg·L^−1^ range (*R*^2^ > 0.999).

#### 3.2.2. Thermogravimetric (TGA) Analysis

TGA was performed using a Mettler Toledo TGA 851e (Mettler Toledo, Giessen, Germany) on samples (5–10 mg) heated at a rate of 20 °C·min^−1^ from 30 °C to 500 °C under a nitrogen atmosphere.

#### 3.2.3. Scanning Electron Microscopy (SEM)

Samples were mounted on aluminum stubs, coated with platinum using an Emitech K550X sputter coater (Emitech, Kent, UK), and surface micrographs were obtained using an F50-FEI (FEI Company, Hillsboro, OR, USA) microscope.

#### 3.2.4. Preparation of MIPs

Typically, MIP preparation was performed in 3.0 mL vials by mixing 0.1 mmol of the template (HCAME), 0.4 mmol of the functional monomer, 2.0 mmol of the crosslinker, and 1% *w*/*w* of the initiator (AIBN) in 400 μL of a porogen mixture consisting of ACN/DMSO (3:1). The standard molar ratio of template to functional monomer to crosslinker was 1:4:20, except when variations in the monomer-to-template ratio were specifically investigated. The mixture was then degassed with nitrogen for 2 min, sealed, and subjected to thermal polymerization at 60 °C for 24 h. To remove the template, unreacted monomers, and crosslinker, a series of washing steps were performed using 5.0 mL × 10 of MeOH/AA (90:10), 5.0 mL × 5 of H_2_O, and 5.0 mL × 5 of MeOH, until no template was detected in the washing solution. The presence of the template was monitored spectrophotometrically using a method described previously [37]. Briefly, 1.8 mL of H_2_SO_4_ 96% (*w*/*w*) was poured into Eppendorf vials containing 0.2 mL of the washing template solution in an ice/water bath. After mixing, the Eppendorf vials were heated to 70 °C for 30 min, and then the absorbance was measured at 389 nm. The resulting hard bulk polymers were then dried, crushed, and ground into a fine powder using a mortar, and sieved (ASTM E11-13 No. 200 and 240 [38]) to obtain particle size fractions between 32 and 45 μm. Particles ≥ 45 μm were further crushed and sieved again. Sedimentation in methanol was used to remove remaining fine particles. Finally, the polymers were dried at 50 °C for 24 h and stored at room temperature for subsequent experiments. MIPs containing styrene were prepared following the same procedure as described above, with styrene added in varying amounts at the expense of the crosslinker, such that the total combined amount of styrene and crosslinker remained constant at 2.0 mmol. The reference non-imprinted polymer (NIP) was prepared using the same procedure, but without the addition of the template molecule. MIPs prepared for studying the effect of the functional monomer were synthesized using the same ratios as described above but on a smaller scale. After polymerization and template removal, the resulting bulk polymers were dried, crushed, and ground into powder and used without further sieving.

#### 3.2.5. UV–Visible Titration Experiments

A series of solutions containing 1 mM VD3 and varying concentrations of 4VP were prepared in MeOH:H_2_O (80:20), and their difference UV absorption spectra were recorded using the corresponding monomer solutions as references with a UV-Vis microplate reader spectrophotometer (Thermo Scientific Fisher, Waltham, MA, USA).

#### 3.2.6. Binding Experiments

Due to the limited aqueous solubility of VD3, MeOH/H_2_O mixture was employed as the medium for binding experiments. Various solvent compositions, ranging from 100% to 80% MeOH (by volume), were tested with both MIP-4VP-DVP and NIP-4VP-DVB to identify the optimal binding medium, which was found to be MeOH/H_2_O (80:20, *v*/*v*). In a typical procedure, 1.0 mM VD3 in MeOH/H_2_O was added to amber vials containing 10.0 mg of polymer, followed by shaking at 50 rpm for 24 h. The mixtures were then filtered through a 0.2 μm cellulose acetate membrane, and the filtrates were analyzed using HPLC-DAD at 285 nm, as previously described.

#### 3.2.7. Adsorption Isotherms

Adsorption isotherms for VD3 were conducted as follows: various concentrations of VD3 in MeOH/H_2_O (80:20, *v*/*v*), ranging from 0.05 to 2.00 mmol/L, were added to Eppendorf vials containing 5.0 mg of polymer. The mixtures were shaken for 24 h at 25 °C, then centrifuged at 5000 rpm for 5 min. The resulting supernatants were analyzed for VD3 content using an HPLC method. The adsorption capacity *Q* (mmol·g^−1^) at each concentration was estimated using the equation:

$$Q=\frac{\left(C_{i}-C_{f}\right)V_{s}}{m_{p}}$$

where *C_i_* and *C_f_* (mmol·L^−1^) are the initial and final concentrations of VD3, respectively, *V_s_* (L) is the volume of the solution and *m_p_* (g) is the mass of the polymer.

### 3.3. Computational Methods

Several initial geometries of four 4VP molecules surrounding HCAME were generated randomly. Each geometry was then energy-minimized employing AMBER force field using the conjugate gradient algorithm (0.01 kcal·mol^−1^·Å^−1^ gradient), followed by further optimization using a semiempirical PM3 method implemented in Gausian16 [39]. The most stable orientation of the 4VP molecules was used for molecular docking studies to find the top-scoring structure of VD3 within the cavity formed by the 4VP molecules. Molecular docking was performed using Autodock Vina 1.2 [40] employing a simulation cube with a length of 25 Å and an exhaustiveness parameter set to 200, while default values were used for the other parameters.

## 4. Conclusions

This work demonstrates the successful synthesis of molecularly imprinted polymers selective toward VD3 using HCAME as a template. Among the various monomers tested, the 4VP–MIP showed the best balance of binding affinity and imprinting factor, which was further enhanced by the introduction of styrene as a co-monomer. Computational and spectroscopic studies confirmed the interactions between VD3 and the 4VP monomer, which are responsible for its recognition. Binding isotherm analysis revealed adsorption consistent with heterogeneous surface interactions, as the data were slightly better fitted by the Freundlich model. TGA and SEM studies revealed that the addition of styrene affects both stability and porosity. These findings highlight the potential of the prepared MIPs as selective sorbents for vitamin VD3 extraction and quantification, addressing current limitations in analytical methods. Future work is needed to evaluate the selectivity of the prepared MIPs toward vitamin D_3_ in the presence of its metabolites.

## Figures and Tables

**Table 1:** Model fitting parameters derived from the binding isotherms of VD3 with MIP-4VP-30%STY and its corresponding NIP.

Polymer	Langmuir					Freundlich				
	Q_max_ (μmol·g^−1^)	K_L_ (L·mmol^−1^)	R^2^	RSS ^a^	F ^b^	*n*	K_F_ (L·mmol^−1^)	R^2^	RSS	F
MIP-4VP-30%STY	385.4	0.61	0.9782	36	37	1.352	148.4	0.9880	134	67
NIP-4VP-30%STY	173.5	0.99	0.9432	43	14	1.145	87.7	0.9689	193	21

^a^ RRS: sum of square of differences between experimental and theoretical data, where the weight for each data point for the non-linear regression was considered as 1/(Q_exp,i_)^2^, (Q_exp,i_ is the experimentally estimated concentration of adsorbed substrates at each mobile phase concentration of substrate). ^b^ F was calculated using the following expression: $F=\frac{\left(n-l\right)\sum_{i=1}^{n}{\left(q_{ex,i}-{\bar{q}}_{ex}\right)}^{2}}{\left(n-1\right)\sum_{i=1}^{n}{\left(q_{ex,i}-q_{t,i}\right)}^{2}}$, where *n* is the number of data points, *l* is the number of parameters, q_ex_ and q_t,i_ are the experimental and theoretical adsorption values, respectively, and ${\bar{q}}_{ex}$ is the mean value of q_ex_.

**Table 2:** Autodock Vina and PM3 estimates of the interaction energies (in kcal·mol^−1^) of the HCAME and VD3 molecules with the surrounding 4VP molecules.

	HCAME	VD3
Autodock Vina	−6.1	−5.7
PM3	−8.8	−6.8

## Data Availability

The original contributions presented in this study are included in the article. Further inquiries can be directed to the corresponding author.
